# The Hidden Burden of Sexual Dysfunction and Healthcare Service Gaps in Tunisian Spinal Cord Patients: A Cross-Sectional Study

**DOI:** 10.3390/jcm14238380

**Published:** 2025-11-26

**Authors:** Ines Loubiri, Ismail Dergaa, Habib Hajji, Halil İbrahim Ceylan, Mariem Gaddour, Nourhene Dridi, Hela Ghali, Valentina Stefanica, Sonia Jemni

**Affiliations:** 1 Faculty of Medicine of Sousse, University of Sousse, Sousse 4000, Tunisia; loubiriiness@famso.u-sousse.tn (I.L.); habib2197@gmail.com (H.H.); gadmariem@gmail.com (M.G.); nourhenedridi498@gmail.com (N.D.); sonia.jemni@famso.u-sousse.tn (S.J.); 2 Physical Medicine and Rehabilitation Department, University Hospital Sahloul, Sousse 4054, Tunisia; hela.ghali@famso.u-sousse.tn; 3 High Institute of Sport and Physical Education of Ksar Said, University of Manouba, Manouba 2010, Tunisia; phd.dergaa@gmail.com; 4 Department of Physical Education of Sports Teaching, Faculty of Sports Sciences, Atatürk University, 25240 Erzurum, Türkiye; 5 Department of Preventive and Community Medicine, University Hospital Sahloul, Sousse 4054, Tunisia; 6 Department of Physical Education and Sport, Faculty of Sciences, Physical Education and Informatics, National University of Science and Technology Politehnica Bucharest, Pitesti University Center, 060042 Pitesti, Romania

**Keywords:** cross-sectional study, erectile dysfunction, female sexual dysfunction, healthcare communication, quality of life, rehabilitation, sexual health, spinal cord injury, Tunisia, urinary incontinence

## Abstract

**Background/Objectives**: Spinal cord injury represents a devastating neurological condition affecting approximately 27 million individuals globally, with particularly severe impacts on sexual function. Sexual dysfunction in SCI patients is multifactorial, with prevalence rates reaching 80–90% across different populations. In low- and middle-income countries, sexual health remains critically neglected in rehabilitation settings due to cultural barriers, inadequate healthcare infrastructure, and limited clinician training. Tunisia, with an estimated 31,000 SCI cases, lacks comprehensive data on sexual dysfunction prevalence and healthcare communication patterns in this vulnerable population. Based on identified research gaps, our study aimed to (i) assess the prevalence and severity of sexual dysfunction among Tunisian SCI patients using validated assessment tools, (ii) identify clinical and demographic factors associated with sexual dysfunction, and (iii) evaluate the current state of sexual health communication and rehabilitation services. **Methods**: A cross-sectional observational study was conducted at Sahloul University Hospital, Sousse, Tunisia, between July and September 2025. Fifty-one adults with chronic SCI (≥12 months post-injury) were recruited through consecutive sampling. Sexual function was assessed using the International Index of Erectile Function (IIEF) for men and the Female Sexual Function Index (FSFI) for women. Additional assessments included demographic data, injury characteristics using the American Spinal Injury Association Impairment Scale, pain evaluation, functional status, psychological well-being, and sleep quality. Statistical analysis included descriptive statistics, Spearman correlations, and significance testing (*p* < 0.05). **Results**: Sexual dysfunction affected 84.3% of participants (43/51), with 88.5% of men experiencing moderate-to-severe erectile dysfunction (median IIEF: 12 [7–36.25]) and 80% of women reporting sexual dysfunction (median FSFI: 7.2 [4–24.25]). Severe dysfunction (FSFI ≤ 10) was present in 56% of female participants. Sexual dysfunction correlated significantly with urinary incontinence (*p* = 0.045) and with measures of functional independence, including SCIM-III (ρ = 0.466, *p* = 0.016) and FIM (ρ = 0.569, *p* = 0.002) among men, and SCIM-III (ρ = 0.469, *p* = 0.018) and FIM (ρ = 0.495, *p* = 0.012) among women, indicating moderate positive associations between sexual and functional outcomes. Only 11.5% of men achieved normal erectile function (IIEF ≥48). Psychological factors (HAD-S) and pain scores (NRS, DN4) were not significantly associated with sexual function. A total of 92% of patients reported never discussing sexual health with their healthcare providers, and 100% lacked access to dedicated sexual rehabilitation services, underscoring severe care and communication gaps in the Tunisian SCI rehabilitation system. **Conclusions**: Sexual dysfunction is highly prevalent among Tunisian spinal cord injury patients and is closely associated with reduced functional independence and urinary issues. Despite its significant impact, sexual health remains largely neglected in rehabilitation care. These findings highlight an urgent need to integrate sexual health into national rehabilitation protocols through provider training, multidisciplinary services, and culturally sensitive education. Systematic sexual health assessment and rehabilitation should be considered essential to improving the quality of life and restoring dignity for affected individuals.

## 1. Introduction

Spinal cord injury represents one of the most significant neurological conditions affecting human health, with a global incidence estimated at 40 to 80 new cases per million people annually, translating to approximately 250,000 to 500,000 new cases worldwide each year [1,2,3,4]. The condition predominantly affects young adults aged 16–40 years, with males constituting approximately 75% of cases and traumatic injuries accounting for 70% of all SCI cases, contributing over 4.5 million years lived with disability annually [1,2].

Regional variations reflect profound differences in healthcare infrastructure, trauma prevention measures, and rehabilitation services, with low and middle-income countries (LMICs) demonstrating concerning patterns of systematic underreporting that likely mask the actual burden due to fragmented healthcare systems [5,6]. The economic impact extends beyond individual suffering, with lifetime costs exceeding $1.5 million per case in developed countries, while LMICs struggle with inadequate resources for comprehensive care delivery [5,7,8].

Sexual dysfunction following SCI represents a multifactorial condition involving complex physiological, psychological, and social factors that profoundly impact quality of life and interpersonal relationships [9,10]. The neurophysiology of sexual function depends on intricate coordination between the autonomic and somatic nervous systems, with specific neural pathways mediating different aspects of sexual response through complex interactions involving sympathetic, parasympathetic, and somatic innervation [10,11]. In men, injuries above T10 typically preserve reflexogenic erections through intact sacral parasympathetic pathways (S2–S4). In contrast, psychogenic erections, mediated by sympathetic thoracolumbar pathways (T11-L2), are commonly compromised, resulting in varied dysfunction patterns depending on the lesion level and completeness [12,13]. Women with SCI frequently experience reduced vaginal lubrication, anorgasmia, and dyspareunia due to disruption of both sympathetic and parasympathetic innervation, with additional complications from autonomic dysfunction affecting cardiovascular responses and genital blood flow [14,15]. The prevalence of sexual dysfunction is consistently high across populations, with studies demonstrating dysfunction rates of 80–95% among men and 70–85% among women, representing one of the most universal consequences of SCI regardless of cultural or geographic context [10].

Despite the overwhelming prevalence and devastating impact on quality of life, sexual health remains critically marginalized within SCI rehabilitation programs worldwide, with particularly severe neglect in LMICs, where cultural stigma, limited healthcare resources, and inadequate provider training compound existing challenges [16,17]. Healthcare communication barriers represent perhaps the most significant obstacle, with studies consistently demonstrating that 70–95% of patients report never discussing sexual concerns with healthcare providers during rehabilitation, stemming from provider discomfort, lack of training, time constraints, cultural taboos, and absence of structured protocols for sexual health screening [18,19]. These communication gaps are exacerbated in traditional societies where sexual topics remain highly stigmatized, creating additional barriers for both patients and healthcare providers who lack culturally appropriate tools and training for addressing sexual health concerns [20,21,22]. The situation in Tunisia and the broader North African region exemplifies these global challenges, compounded by conservative cultural attitudes, healthcare system limitations, and the complete absence of specialized sexual medicine services within public healthcare infrastructure [23,24].

Based on the identified research gaps in sexual dysfunction prevalence, associated factors, and healthcare service delivery patterns in the Tunisian SCI population [25,26,27], our study aimed to (i) assess the prevalence and severity of sexual dysfunction among Tunisian SCI patients using validated assessment tools, (ii) identify clinical and demographic factors associated with sexual dysfunction, and (iii) evaluate the current state of sexual health communication and rehabilitation services in this underserved population.

## 2. Materials and Methods

### 2.1. Ethical Approval

The study received approval from the Ethical Committee of Sahloul Hospital (approval number: HS41-2025, 12 June 2025) and was conducted in accordance with the principles of the Declaration of Helsinki. Verbal informed consent was obtained from all patients before participation through telephone interviews with particular attention to confidentiality, given the sensitive nature of sexual health assessments.

### 2.2. Sample Size Calculation

Sample size was determined using the standard formula for cross-sectional prevalence studies: n = Z^2^ × p(1 − p)/d^2^, where Z = 1.96 (95% confidence level), p is the expected prevalence, and d is the desired precision. An expected prevalence of *p* = 0.80 was selected based on international evidence: Cobo-Cuenca et al. (2017) reported 80–95% prevalence in male SCI patients [28], Di Giusto et al. (2023) documented 70–85% in Latin American populations [29], and the Fifth International Consultation on Sexual Medicine (ICSM, 2024) confirmed 80–90% prevalence across diverse SCI populations [10].

The desired precision was set at d = 0.11, reflecting the balance between methodological rigor and recruitment feasibility. While this exceeds conventional thresholds, it aligns with the guidance of Pourhoseingholi et al. [30], who suggest that precision values may reach 0.10 or higher in high-prevalence conditions (*p* ≥ 0.6). The marginally wider precision was necessitated by limited eligible patients in Tunisia’s two national rehabilitation centers, cultural barriers to discussing sexual health, and the absence of prior national data.

Calculation:                          n = (1.96)^2^ × 0.80(1 − 0.80)/(0.11)^2^ = 3.8416 × 0.16/0.0121 = 50.8 ≈ 51

Post hoc verification: With our achieved sample (n = 51) and observed prevalence of 84.3%, the 95% confidence interval is [71.8%, 92.2%], yielding an achieved precision of ±10.2%, which closely approximates our target and confirms sample adequacy. This sample size is consistent with established SCI studies: Phelps et al. (n = 50) [31], Tzanos et al. (n = 30) [32], and Salmani et al. (n = 28) [33].

### 2.3. Population

The study included adults aged ≥18 years with chronic SCI, defined as disease duration ≥ 12 months post-injury, who provided verbal consent. Participants were recruited from the Physical Medicine and Rehabilitation inpatient department at Sousse, Tunisia, which admits patients from multiple regions across the central and southern regions. Exclusion criteria comprised concurrent neurological disorders potentially affecting sexual function (Parkinson’s disease, traumatic brain injury, stroke, multiple sclerosis) and current psychiatric disorders such as schizophrenia, delusional states, severe depression, and severe anxiety requiring active treatment, to ensure observed effects could be attributed specifically to SCI.

### 2.4. Experimental Design

This cross-sectional, observational, analytical study was conducted at the Department of Physical Medicine and Rehabilitation, Sahloul University Hospital (Sousse, Tunisia) between July and September 2025. Admitted Patient records from the 2024 inpatient rehabilitation unit were retrospectively reviewed for eligibility. Eligible patients were consecutively selected and contacted via telephone for a sexual function assessment conducted by two trained operators to ensure consistency and minimize interviewer bias. Standardized interview protocols were implemented to maintain data quality across assessments, with comprehensive training provided to reduce inter-observer variability in discussions of sensitive health topics.

### 2.5. Assessment Tools

#### 2.5.1. Demographic and Clinical Assessment

A comprehensive standardized form captured demographic variables, including age, gender, marital status, educational level, and employment status. Clinical variables included time since injury, etiology of SCI, neurological level of injury, and American Spinal Injury Association Impairment Scale (AIS) classification [34]. The AIS provides a standardized assessment of motor, sensory, and sacral function with five grades (A–E) and serves as the gold standard for documenting the neurological level and severity of SCI.

#### 2.5.2. Pain Assessment Tools

Pain evaluation utilized two validated instruments: the Numerical Rating Scale (NRS) for nociceptive pain assessment, scored from 0 to 10 with higher scores indicating greater pain intensity, and the Douleur Neuropathique 4 (DN4) questionnaire for neuropathic pain screening [35]. The DN4 consists of 10 items with a cut-off score of 4/10 for neuropathic pain diagnosis, demonstrating sensitivity of 82.9% and specificity of 89.9% in neurological populations.

#### 2.5.3. Functional Independence Assessment

Functional status was assessed using two complementary instruments. The Functional Independence Measure (FIM) evaluates independence in self-care, sphincter control, transfers, locomotion, communication, and social cognition across 18 items [36]. Each item is scored from 1 (total assistance) to 7 (complete independence), yielding total scores ranging from 18 to 126, with higher scores indicating greater independence. The Spinal Cord Independence Measure version III (SCIM-III) assesses explicitly the independence of SCI patients across 19 items in three domains: self-care (0–20 points), respiration and sphincter management (0–40 points), and mobility (0–40 points) [37]. Total scores range from 0 to 100, with higher scores indicating greater functional independence.

#### 2.5.4. Psychological Status Assessment

The Hospital Anxiety and Depression Scale (HAD-S) assessed psychological well-being through 14 self-report items: 7 for anxiety and 7 for depression subscales [38]. Each subscale scores 0–21, with higher scores indicating greater symptom severity. The HAD-S demonstrates excellent internal consistency in SCI populations and has been validated in 963 community-dwelling individuals with SCI [39]. Cut-off scores of 8 or higher provide optimal sensitivity and specificity for screening, while scores of 11 or higher indicate clinically significant symptoms.

#### 2.5.5. Sleep Quality Evaluation

The Pittsburgh Sleep Quality Index (PSQI) assessed sleep quality and disturbances over the past month using 19 questions grouped into seven components [40]. Components include subjective sleep quality, sleep latency, sleep duration, habitual sleep efficiency, sleep disturbances, use of sleeping medication, and daytime dysfunction. Each component scores from 0 (no difficulty) to 3 (severe difficulty), with total scores ranging from 0 to 21. A global score > 5 indicates poor sleep quality.

#### 2.5.6. Male Sexual Function Assessment Questionnaire

The International Index of Erectile Function (IIEF) assessed male sexual function across five domains: erectile function (6 items, range 1–30), orgasmic function (2 items, range 0–10), sexual desire (2 items, range 2–10), intercourse satisfaction (3 items, range 0–15), and overall satisfaction (2 items, range 2–10). The IIEF exhibits excellent psychometric properties, characterized by high internal consistency, test–retest reliability, and sensitivity to treatment effects across diverse populations [41]. Total scores range from 5 to 75, with established cutoffs: normal function ≥48, mild dysfunction 37–47, and moderate/severe dysfunction ≤36. The French version was translated into Arabic by a qualified physician to ensure linguistic accessibility; however, this translated version has not undergone formal validation.

#### 2.5.7. Female Sexual Function Assessment Questionnaire

The Female Sexual Function Index (FSFI) evaluated female sexual function through 19 items across six domains: desire (2 items), arousal (4 items), lubrication (4 items), orgasm (3 items), satisfaction (3 items), and pain (3 items). Each domain receives a weighted score, with the composite score ranging from 2 to 36; higher scores indicate better sexual function. The FSFI was administered in its validated Arabic version, previously adapted for the Egyptian population [42], demonstrating excellent reliability (Cronbach’s α = 0.97) and construct validity. Established cut-offs classify sexual function as: normal ≥26.55, mild risk 18–26.55, moderate risk 11–17, and severe dysfunction <10.

#### 2.5.8. Sexual Health Communication Assessment

Sexual health communication and service access were evaluated through two structured questions: “Has your attending physician ever discussed sexual health with you during your rehabilitation?” and “Have you ever received sexual rehabilitation services or counselling?” Both questions utilized a yes/no response format to assess current gaps in healthcare delivery and communication patterns.

### 2.6. Statistical Analysis

Statistical analysis was performed using SPSS version 25.0. Normal distribution was assessed using the Kolmogorov–Smirnov test. Parameters with normal distribution were expressed as means and standard deviations (SD), while non-normally distributed parameters were expressed as medians and interquartile ranges [IQR]. Categorical variables were summarized using frequencies and percentages. Univariate comparisons between patients with and without sexual dysfunction were performed using appropriate tests based on variable type and distribution. For categorical variables, the Pearson Chi-Square test or exact Fisher test was used. For continuous variables, the Independent Samples *t*-test was used if the data met assumptions of normality; otherwise, the Mann–Whitney U test was applied.

In the multivariate analysis, variables with *p*-values < 0.05 were included in a binary logistic regression model. Variables with *p* < 0.05 after multivariate analysis were considered independent factors of sexual dysfunction. Adjusted odds ratios (OR) and their 95% confidence intervals (CI) were calculated to estimate the strength of the association. Correlation analyses were conducted using Spearman’s rank correlation coefficients, appropriate for non-normally distributed data, to examine relationships between clinical variables and sexual function scores. The strength of the correlations was categorized as follows: very strong (≥0.70), strong (0.50–0.69), moderate (0.30–0.49), and weak (<0.30). A *p*-value less than 0.05 was considered statistically significant [43].

### 2.7. Use of GenAI in Writing

In preparing this paper, the authors utilized the ChatGPT model 4 on 18 September 2025, to revise specific passages of the manuscript, double-check for grammatical errors, and enhance academic English. After using this tool, the authors have reviewed and edited the content as necessary and take full responsibility for the content of the publication.

## 3. Results

### 3.1. Demographic and Clinical Characteristics

**Figure 1:**
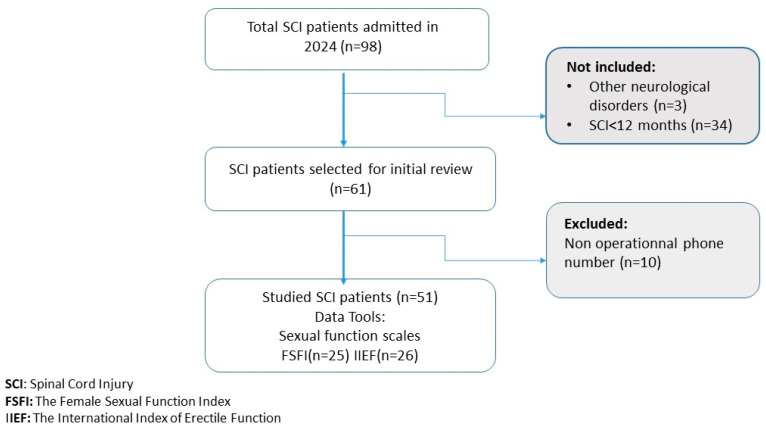
Flow chart of the study population.

Fifty-one patients were enrolled in this study, as shown in the flowchart in Figure 1. The study population had a mean age of 43.47 ± 15.43 years and demonstrated a near-equal sex distribution, with a male-to-female ratio of 1.04. The majority of participants were married (64.7%) and had medium socioeconomic status (60.8%). Comorbid conditions were present in 11.8% of participants, with diabetes mellitus being the most prevalent (7.8%), followed by hypertension (5.9%). Table 1 presents the complete sociodemographic characteristics of the study population, including the distribution of educational levels and employment status across the cohort.

### 3.2. Injury Characteristics and Clinical Profile

Injury characteristics revealed a median time since injury of 22 months [13:36], with traumatic etiology predominating (72.5%). Neurological assessment showed 29.4% with complete injuries (AIS A), 11.8% with sensory incomplete (AIS B), 31.4% with motor incomplete (AIS C), and 27.5% with preserved motor function (AIS D). Cervical injuries accounted for 43.1%, thoracic injuries 37.3%, and lumbar injuries 19.6% of cases. Table 2 presents detailed injury characteristics, including etiological distribution and neurological classification patterns across male and female participants.

### 3.3. Pain Assessment and Complications

Pain assessment revealed heterogeneous patterns across the cohort. Median NRS score was 4 [2–6], indicating moderate pain levels, while median DN4 score was 3 [2–5]. Nociceptive pain predominantly manifested as shoulder pain affecting 43.1% of participants, while neuropathic pain occurred at the lesion level in 21.5% and below the lesion level in 52.9% of cases. Cutaneous complications were observed in 23.5% of patients, with pressure ulcers distributed across different stages. Neurogenic heterotopic ossifications occurred in 17.6% of cases, primarily affecting the hips (11.8%) and knees (7.8%). Bladder and bowel dysfunction were prevalent, with urinary incontinence reported in 31.4% of cases and constipation in 44% of cases.

### 3.4. Functional Status Assessment

Functional assessment demonstrated significant variability in independence levels. Mean SCIM-III score was 56.24 ± 21.8, while mean FIM score was 76.18 ± 22.0, indicating moderate functional independence across the cohort. SCIM-III subdomain analysis revealed self-care scores of 12.4 ± 5.8, respiration and sphincter management scores of 23.6 ± 9.4, and mobility scores of 20.2 ± 8.9. 

### 3.5. Psychological and Sleep Quality Assessment

Psychological evaluation using HAD-S revealed a median anxiety subscale score of 6 [5–9] and a median depression subscale score of 7 [4–9]. Only 5.9% of patients exhibited clinically significant anxiety or depression symptoms (HAD-S ≥ 11). Sleep quality assessment showed a median PSQI score of 7 [5–8], with 80.4% of patients reporting poor sleep quality (PSQI > 5). 

### 3.6. Male Sexual Function Assessment

Among male patients (n = 26), the median IIEF total score was 12 [7–36.25], indicating moderate-to-severe sexual dysfunction. Table 3 presents the comprehensive IIEF domain scores and dysfunction severity classifications for the male cohort, along with detailed descriptive statistics for each domain.

### 3.7. Female Sexual Function Assessment

Among female patients (n = 25), 80% reported sexual dysfunction with a median FSFI total score of 7.2 [4–24.25]. Domain-specific analysis revealed profound impairment across all dimensions. Severe dysfunction (FSFI ≤ 10) affected 56% of women, while moderate dysfunction (FSFI 11–17) affected 24%. Table 4 represents the detailed FSFI domain scores and dysfunction classifications demonstrating the pervasive nature of sexual impairment in this population.

### 3.8. Associated Factors with Sexual Dysfunction: Univariate and Multivariate Analysis

#### 3.8.1. Univariate Analysis

Table 5 represents univariate analysis of sociodemographic, clinical, complications, functional, psychological, and sleep quality outcomes, showing significant correlations with urinary incontinence (*p* = 0.045), SCIM-III scores (*p* = 0.010), and FIM scores (*p* = 0.011).

#### 3.8.2. Multivariate Analysis

In the multivariate analysis, the independent factor associated with sexual dysfunction was the SCIM-III score, with a *p*-value of 0.026 and an adjusted odds ratio (ORa) of 0.944 [95% CI: 0.897–0.993].

#### 3.8.3. Correlation Analyses

Table 6 and Table 7 present detailed correlation analyses for IIEF and FSFI scores, respectively, with clinical variables, function scores, and functional independence measures. Spearman’s correlation analysis revealed positive, significant associations between sexual dysfunction, urinary incontinence, and functional outcomes.

**Figure 2:**
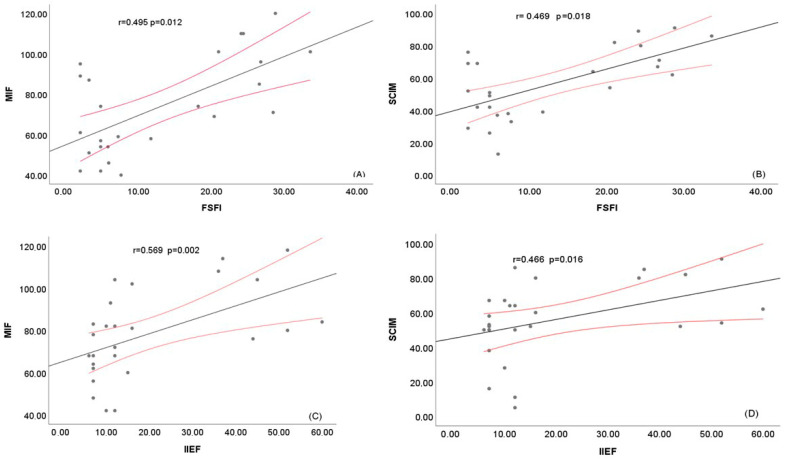
Spearman Correlation Scatter Plot Analysis Between Functional and Sexual Health Scores. Scatter plots illustrate the relationships between sexual health scores (FSFI, IIEF) and functional assessment scores (FIM, SCIM). Each plot includes a fitted regression line (black), 95% confidence intervals (red), and Spearman correlation coefficients (r) with associated *p*-values. (**A**) FSFI vs. FIM: Moderate positive correlation (r = 0.495, *p* = 0.012). (**B**) FSFI vs. SCIM: Moderate positive correlation (r = 0.469, *p* = 0.018). (**C**) IIEF vs. FIM: Strong positive correlation (r = 0.569, *p* = 0.002). (**D**) IIEF vs. SCIM: Moderate positive correlation (r = 0.466, *p* = 0.016).

Figure 2 represents scatter plots illustrating the relationships between sexual dysfunction and functional outcomes. 

### 3.9. Sexual Health Communication and Service Access

When asked, “Has your attending physician ever discussed sexual health with you?”, 92% of patients (n = 47/51) reported that clinicians never initiated discussions about sexual health during routine follow-ups or rehabilitation encounters. The question “Have you ever received sexual rehabilitation services?” revealed that 100% of participants lacked access to specialized sexual rehabilitation services, counseling, or structured interventions due to the complete absence of such programs within Tunisia’s public healthcare system.

## 4. Discussion

This study reveals a high prevalence of sexual dysfunction among Tunisian patients with spinal cord injury (SCI), affecting 84.3% of participants. The findings confirm our initial hypothesis and highlight more extensive gaps in sexual health services than anticipated, including a complete absence of specialized care and significant communication barriers between patients and providers.

Among male participants, the median International Index of Erectile Function (IIEF) score was 12 [7–36.25], well below the clinical threshold of 48, with 88.5% reporting moderate to severe erectile dysfunction. This prevalence exceeds the 69.2% reported by Migaou et al. in a Tunisian cohort of paraplegic males, likely because we included both paraplegic and tetraplegic patients [23]. Comparable dysfunction rates have been reported internationally, including 89.4% in Spanish SCI males [28], and similar findings have been reported in multicenter European studies [44], supporting the robustness of our results.

The large effect size (Cohen’s d = 1.2) further underscores the clinical significance of these impairments compared to normative populations [45,46]. Female participants also demonstrated severe dysfunction, with a median Female Sexual Function Index (FSFI) score of 7.2 [4–24.25], far below the threshold of 26.55. Notably, 56% scored ≤ 10, indicating profound impairment across all domains, particularly in orgasm (median 2 [0–3.2]) and arousal (median 0.8 [0–4.5]). These results are consistent with international data, including FSFI scores of 14.4 ± 13.7 in Greek women [32] and similar patterns in Iranian SCI patients [47].

The consistency of sexual dysfunction across diverse populations, including comparisons with Tunisian patients with multiple sclerosis [48], suggests that neurophysiological mechanisms underlying sexual impairment in SCI are universal and not confined by cultural or geographic boundaries [49]. These findings reinforce the need to integrate sexual health into rehabilitation protocols as a core component of care. Cultural sensitivity should guide the development of appropriate interventions, but must not justify the exclusion of essential sexual health services. Addressing both medical and psychosocial aspects of sexual dysfunction is critical to improving quality of life and restoring intimate relationships in this population.

This study highlights a critical gap in sexual healthcare among Tunisian patients with spinal cord injury (SCI). Notably, 92% of participants had never discussed sexual health with a healthcare provider, a rate significantly higher than the 65–70% communication gaps reported in other healthcare systems [50]. Furthermore, none of the patients had access to sexual rehabilitation services, reflecting broader systemic challenges in LMICs, where sexual health is often deprioritized due to limited resources, insufficient provider training, and cultural constraints. Compared to high-income countries, where 40–60% of SCI patients receive some form of sexual health counseling, this disparity represents a large effect size (Cohen’s d > 2.0), underscoring inequities in care that cannot be justified solely by cultural norms [51].

Encouragingly, programs such as the sexual and reproductive health initiative supported by the International Development Research Centre (IDRC) in Tunisia demonstrate the feasibility of integrating sexual health into clinical services through multidisciplinary collaboration and culturally sensitive education. These models may inform the development of structured sexual rehabilitation protocols tailored to the Tunisian context [52].

To address these gaps, healthcare providers require formal training, standardized screening tools, and institutional support to deliver evidence-based sexual health interventions. System-level reforms should prioritize integrating sexual health into provider education, policy development, and resource allocation.

Our findings also reveal moderate correlations between sexual dysfunction and functional independence, as measured by SCIM-III (r = 0.34, *p* = 0.016) and FIM (r = 0.35, *p* = 0.011). These associations suggest that patients with greater mobility and autonomy may experience better sexual outcomes, potentially due to improved positioning, reduced caregiver dependence, and enhanced self-esteem. However, these correlations explain only 11–12% of the variance in sexual function, indicating that functional status alone is insufficient to predict sexual health [53]. Similar findings have been reported by Di Giusto et al. (r = 0.42) [29] and Harrison et al. (r = 0.38), reinforcing the need for a multidisciplinary approach that combines functional rehabilitation with medical, psychological, and relational support [54].

Sexual dysfunction in SCI is multifactorial, involving both neurophysiological and psychosocial mechanisms. Damage to spinal pathways affecting autonomic regulation and sensory-motor integration can impair arousal, genital response, and orgasm. Studies report that 70–81% of men experience erectile dysfunction, nearly all male patients have ejaculation difficulties, and 50–80% of women report reduced arousal or vaginal dryness. Psychosocial factors such as altered body image, emotional distress, and relationship challenges further exacerbate sexual difficulties and may persist despite physical recovery [10,55].

Urinary incontinence also showed a significant correlation with sexual dysfunction (r = −0.29, *p* = 0.045), reflecting shared autonomic pathways and psychological impacts. Concerns about hygiene, embarrassment, and spontaneity during intimacy may contribute to reduced sexual confidence [56,57]. Similar associations have been documented by Cramp et al. (r = −0.31) [56] and Sramkova et al. [58]. These findings highlight the importance of integrating urological management into sexual rehabilitation, including catheter education, positioning strategies, and individualized care plans.

Contrary to expectations, pain, depression, and sleep disturbances showed limited associations with sexual dysfunction. Only anxiety scores on the HAD-S scale correlated with specific IIEF domains (orgasmic function: r = −0.31, *p* = 0.043; intercourse satisfaction: r = −0.29, *p* = 0.056), representing small effect sizes. This contrasts with stronger correlations reported in other populations, such as those by Pakpour et al. in Iranian SCI patients [59]. The weaker associations in our cohort may reflect cultural differences in symptom reporting, low prevalence of psychological distress (5.9%), or potential resilience factors. Alternatively, the cross-sectional design may not capture evolving psychological adaptation over time.

Future research should explore cultural influences on psychological resilience and their impact on sexual recovery. Despite limited statistical associations, psychological support remains a vital component of comprehensive sexual rehabilitation and should be systematically included in care protocols.

### Limitations

This pioneering study provides the first comprehensive evaluation of sexual function among Tunisian SCI patients of both genders, establishing essential baseline data and identifying critical clinical correlates. However, several methodological limitations must be acknowledged. This study’s small sample size (n = 51) limits statistical power and generalizability, although it remains consistent with similar single-center research. The use of telephone-administered sexual health questionnaires, while necessary due to logistical and cultural factors, may have introduced social desirability bias. Additionally, non-response to phone contact excluded several eligible participants, potentially leading to selection bias by over-representing individuals who are more socially engaged or motivated, thereby affecting the representativeness of the findings. However, this sample size aligns with those reported in similar single-center studies conducted in comparable clinical and cultural contexts. For example, Migaou et al. studied 30 Tunisian males [23], Tzanos et al. studied 30 Greek women [32], and Hajiaghababaei et al. examined 105 Iranian women [47]. The cross-sectional design precludes the determination of causal relationships and temporal sequences, limiting understanding of how sexual function evolves during rehabilitation. The absence of control groups prevents comparison with general population sexual function norms, though published normative data provide some reference points. Selection bias may have occurred due to consecutive sampling from a single rehabilitation center, potentially over-representing more severe cases or those with better access to healthcare. The study did not include healthcare provider perspectives, limiting understanding of the systemic factors contributing to communication barriers and service gaps.

## 5. Conclusions

Sexual dysfunction among Tunisian spinal cord injury patients, affecting over 84% of the sample, represents a critical yet underrecognized healthcare need. Significant associations with functional independence (SCIM-III, FIM scores) and urological complications (urinary incontinence) highlight the multifactorial nature of sexual health challenges and provide specific targets for comprehensive intervention development that must address both neurological and psychosocial factors. Integrating sexual health into rehabilitation policy is essential for improving patient outcomes and promoting equitable care. A strategic model could include routine screening, multidisciplinary collaboration, provider education, and national guidelines supported by institutional frameworks. This approach aligns with international initiatives, such as the WHO Rehabilitation 2030 agenda, which advocates for person-centered, inclusive rehabilitation. Recognizing sexual health as a core component of recovery can enhance quality of life and ensure comprehensive, responsive healthcare delivery.

## Figures and Tables

**Table 1:** Sociodemographic characteristics of the population.

Characteristics	Total Population	Male Patients (n = 26)	Female Patients (n = 25)
Age, years, Mean ± SD	43.47 ± 15.43	42.04 ± 15.08	44.16 ± 15.86
Marital status, N (%)			
Married	33 (64.7)	15 (57.7)	18 (72)
Single	18 (35.3)	11 (42.3)	7 (28)
Residence, N (%)			
Urban	34 (66.7)	16 (61.5)	18 (72)
Rural	17 (33.3)	10 (38.5)	7 (28)
Socioeconomic level, N (%)			
Low	15 (29.4)	8 (30.8)	7 (28)
Medium	31 (60.8)	14 (53.8)	17 (68)
Good	5 (9.8)	4 (15.4)	1 (4)
Comorbid conditions, N (%)			
Yes	6 (11.8)	4 (15.4)	2(8)
No	45 (88.2)	22 (84.6)	23 (92)
Smoking, N (%)	19 (37.3)	18 (69.2)	1 (4)

**Table 2:** Clinical characteristics of injury.

Characteristics	Population (n = 51)	Male Patients (n = 26)	Female Patients (n = 25)
Time since injury, months, median, [IQR]	22 [13–36]	16 [13–29.25]	29 [18–37]
Etiology, N (%)			
Traumatic	37 (72.5)	18 (69.2)	19(76)
Non-traumatic	14 (27.5)	8 (30.8)	6 (24)
NLI, N (%)			
Cervical	22 (43.1)	11 (42.3)	11 (44)
Dorsal	19 (37.3)	9 (34.6)	10 (40)
Above T10	31 (60.8)	16 (61.5)	15 (60)
Lumbar	10 (19.6)	6 (23.1)	4 (16)
AIS, N (%)			
A	15 (29.4)	8 (30.8)	7 (28)
B	6 (11.8)	4 (15.4)	2 (8)
C	16 (31.4)	8 (30.8)	8 (32)
D	14 (27.5)	6 (23.1)	8 (32)

NLI: neurological level of injury; AIS: ASIA Impairment Scale; IQR: Interquartile range.

**Table 3:** IIEF subscale results.

	N	Median	IQR
Erectile function	26	1	[1–12.25]
Orgasmic function	26	1	[1–6.5]
Sexual desire	26	3.5	[3–5.25]
Intercourse satisfaction	26	3	[0–4.75]
Overall satisfaction	26	2.5	[2–6]
IIEF	26	12	[7–36.25]

IIEF: International Index of Erectile Function, IQR: Interquartile ranges.

**Table 4:** FSFI subscale results.

	Median	IQR
FSFI	7.2	[4–24.25]
FSFI desire	1.2	[1.2–4.5]
FSFI arousal	0.8	[0–4.5]
FSFI lubrication	1.5	[0–4.05]
FSFI orgasm	2	[0–3.2]
FSFI satisfaction	3.6	[1–4.8]
FSFI pain	0	[0–3.6]

FSFI: Female Sexual Function Index; IQR: Interquartile range.

**Table 5:** Univariate analysis of sociodemographic, clinical variables, complications, functional, psychological, and sleep quality outcomes.

Variable	Value	Sexual Dysfunction No (n = 8)	Sexual Dysfunction Yes (n = 43)	*p*-Value
Age Median, SD		40.7 ± 14.8	43.5 ± 15.5	0.645
Marital Status N (%)	Married	6 (18.2)	27 (81.8)	0.696
	Single	2 (11.1)	16 (88.9)	
Residence N (%)	Urban	5 (14.7)	29 (85.3)	1.000
	Rural	3 (17.6)	14 (82.4)	
Socioeconomic level N (%)	Low	4 (26.7)	11 (73.3)	0.213
	Medium,	4 (11.1)	32 (88.9)	
	Good			
Comorbidities N (%)	Yes	-	6 (14)	0.572
Smoking N (%)	yes	2(25)	17(39.5)	0.694
Etiology	Traumatic	5 (13.5)	32 (86.5)	0.668
	Non traumatic	3 (21.4)	11 (78.6)	
Time since injury Median [IQR]		33.5 [13–70.5]	20 [13–36]	0.491
AIS N (%)	complete	**-**	15 (34.9)	0.087
	incomplete	8 (100)	28 (65.1)	
Level D10	Above D10	4 (50)	27 (62.8)	0.696
	Below D10	4 (50)	16 (37.2)	
Urinary Incontinence N (%)		-	16 (37.2)	**0.045**
Pressure ulcers N (%)		1 (12.5)	11 (25.6)	0.662
NHO N (%)		-	9 (20.9)	0.322
NRS [IQR]		4.5 [0.75–5.75]	4 [2–6]	0.895
DN4 [IQR]		3 [1–6.5]	3 [2–5]	0.979
HAD-S A [IQR]		7.5 [6–9]	6 [5–9]	0.183
HAD-S D [IQR]		6.5 [4.5–9.75]	7 [4–9]	0.794
SCIM-III [IQR]		69 [62–89.75]	52 [38–69]	**0.010**
FIM Mean, SD		94.38 ± 17.77	72.79 ± 21.80	**0.011**
PSQI Mean, SD		8.12 ± 5.41	6.79 ± 2.66	0.855

NHO: Neurogenic Heterotopic Ossifications; NRS: Numerical Rating Scale; DN4: Douleur Neuropathique 4; HAD-S: Hospital Anxiety and Depression Scale; A: Anxiety; D: Depression; SCIM-III: Spinal Cord Independence Measure version III; FIM: Functional Independence Measure; PSQI: Pittsburgh Sleep Quality Index; AIS: ASIA Impairment Scale; IQR: Interquartile Ranges.

**Table 6:** Correlation of the International Index of Erectile Function with clinical variables.

		Erectile Function	Orgasmic Function	Sexual Desire	Intercourse Satisfaction	Overall Satisfaction	IIEF Total
Age	*r*	−0.118	−0.21	−0.223	0.012	−0.085	−0.064
	*p*-value	0.565	0.304	0.273	0.955	0.681	0.756
Time since injury	*r*	−0.371	**−0.432**	−0.312	−0.17	−0.151	−0.192
	*p*-value	0.062	**0.027**	0.121	0.405	0.461	0.346
NRS	*r*	−0.263	−0.376	−0.297	−0.274	−0.186	−0.287
	*p*-value	0.194	0.059	0.141	0.176	0.364	0.155
DN4	*r*	−0.245	−0.326	−0.264	−0.355	−0.228	−0.225
	*p*-value	0.228	0.104	0.192	0.075	0.262	0.27
HAD-S A	*r*	0.35	**0.423**	0.333	0.407	0.053	0.303
	*p*-value	0.08	**0.031**	0.097	**0.039**	0.797	0.133
HAD-S D	*r*	−0.038	−0.093	−0.016	0.101	0.088	0.134
	*p*-value	0.855	0.652	0.938	0.623	0.668	0.515
SCIM-III	*r*	**0.529**	**0.457**	0.377	0.129	**0.55**	**0.466**
	*p*-value	**0.005**	**0.019**	0.058	0.531	**0.004**	**0.016**
FIM	*r*	**0.609**	**0.552**	**0.501**	0.29	**0.565**	**0.569**
	*p*-value	**0.001**	**0.003**	**0.009**	0.151	**0.003**	**0.002**
PSQI	*r*	0.028	0.019	−0.267	0.183	−0.083	−0.073
	*p*-value	0.892	0.927	0.188	0.37	0.685	0.724

NRS: numerical rating Scale; DN4: Douleur Neuropathique 4; HAD-S: Hospital Anxiety and Depression Scale; SCIM-III: Spinal Cord Independence Measure Version III; FIM: Functional Independence Measure; PSQI: Pittsburgh Sleep Quality Index; r: Spearman’s rho.

**Table 7:** Correlation between the Female Sexual Function Index and clinical variables.

		Desire	Arousal	Lubrication	Orgasm	Satisfaction	Pain	Total
Age	*r*	0.071	0.085	0.144	0.244	0.045	0.076	0.191
	*p*-value	0.738	0.687	0.491	0.24	0.833	0.716	0.36
Time since injury	*r*	0.45	0.369	0.362	0.32	0.31	0.287	0.39
	*p*-value	**0.024**	0.069	0.075	0.119	0.131	0.165	0.054
NRS	*r*	0.002	0.017	−0.015	0.099	−0.049	−0.116	0.1
	*p*-value	0.993	0.936	0.945	0.638	0.817	0.582	0.635
DN4	*r*	0.162	0.258	0.226	0.274	0.06	0.212	0.183
	*p*-value	0.44	0.214	0.278	0.186	0.774	0.309	0.381
HAD-S anxiety	*r*	0.256	0.261	0.229	0.316	0.21	0.137	0.284
	*p*-value	0.217	0.207	0.27	0.124	0.314	0.513	0.169
HAD-S depression	*r*	−0.027	0.163	0.079	0.227	−0.003	−0.053	0.184
	*p*-value	0.899	0.438	0.706	0.275	0.988	0.802	0.379
SCIM-III	*r*	0.664	0.536	0.564	0.492	0.508	0.683	0.469
	*p*-value	**<0.001**	**0.006**	**0.003**	**0.013**	**0.01**	**<0.001**	**0.018**
FIM	*r*	0.666	0.557	0.589	0.509	0.482	0.648	0.495
	*p*-value	**<0.001**	**0.004**	**0.002**	**0.009**	**0.015**	**<0.001**	**0.012**
PSQI	*r*	−0.164	−0.092	−0.173	−0.022	−0.188	−0.142	−0.097
	*p*-value	0.433	0.662	0.407	0.917	0.369	0.498	0.646

NRS: numerical rating scale; DN4—Douleur Neuropathique 4 Questionnaire; HAD-S—Hospital Anxiety and Depression Scale; SCIM-III—Spinal Cord Independence Measure, version III; FIM—Functional Independence Measure; PSQI—Pittsburgh Sleep Quality Index; FSFI—Female Sexual Function Index; r: Spearman’s rho.

## Data Availability

The authors will make the raw data supporting this article’s conclusions available upon request.
